# A Receptor-Based Explanation for Tsetse Fly Catch Distribution between Coloured Cloth Panels and Flanking Nets

**DOI:** 10.1371/journal.pntd.0004121

**Published:** 2015-10-16

**Authors:** Roger D. Santer

**Affiliations:** Institute of Biological, Environmental, and Rural Sciences, Aberystwyth University, Aberystwyth, Ceredigion, United Kingdom; International Centre of Insect Physiology and Ecology, KENYA

## Abstract

Tsetse flies transmit trypanosomes that cause nagana in cattle, and sleeping sickness in humans. Therefore, optimising visual baits to control tsetse is an important priority. Tsetse are intercepted at visual baits due to their initial attraction to the bait, and their subsequent contact with it due to landing or accidental collision. Attraction is proposed to be driven in part by a chromatic mechanism to which a UV-blue photoreceptor contributes positively, and a UV and a green photoreceptor contribute negatively. Landing responses are elicited by stimuli with low luminance, but many studies also find apparently strong landing responses when stimuli have high UV reflectivity, which would imply that UV wavelengths contribute negatively to attraction at a distance, but positively to landing responses at close range. The strength of landing responses is often judged using the number of tsetse sampled at a cloth panel expressed as a proportion of the combined catch of the cloth panel and a flanking net that samples circling flies. I modelled these data from two previously published field studies, using calculated fly photoreceptor excitations as predictors. I found that the proportion of tsetse caught on the cloth panel increased with an index representing the chromatic mechanism driving attraction, as would be expected if the same mechanism underlay both long- and close-range attraction. However, the proportion of tsetse caught on the cloth panel also increased with excitation of the UV-sensitive R7p photoreceptor, in an apparently separate but interacting behavioural mechanism. This R7p-driven effect resembles the fly open-space response which is believed to underlie their dispersal towards areas of open sky. As such, the proportion of tsetse that contact a cloth panel likely reflects a combination of deliberate landings by potentially host-seeking tsetse, and accidental collisions by those seeking to disperse, with a separate visual mechanism underlying each behaviour.

## Author Summary

Tsetse flies transmit trypanosomes that cause sleeping sickness. Visual baits to attract and kill tsetse are an important method of vector control, and the rational improvement of these baits depends on a mechanistic understanding of tsetse behaviour. Visual baits are often panels of insecticide-treated cloth which tsetse must contact to become dosed with insecticide. However, most of the tsetse that are attracted to approach visual baits circle them rather than landing. Colour is one factor that might be important in eliciting landing responses, and thus bait optimisation. Visually-driven tsetse behaviour can be understood by investigating how a fly’s five types of photoreceptor respond to differently coloured baits, and determining how each of these photoreceptors contributes to behaviour. I applied this approach to data recorded in two previous field studies. I found that tsetse contacted visual baits due to two behavioural mechanisms: a comparison between the responses of several photoreceptors that underlies attraction and landing, and a UV photoreceptor-driven mechanism that likely drives dispersal towards open sky and causes tsetse to collide with visual baits accidentally. If the mechanistic basis of tsetse behaviour is understood, it may be possible to design baits that exploit these mechanisms and optimise tsetse control.

## Introduction

Tsetse flies (*Glossina* spp.) occur in sub-Saharan Africa and transmit the trypanosomes that cause nagana in cattle, and sleeping sickness (human African trypanosomiasis, HAT) in humans [1]. Riverine tsetse (Palpalis species group) are responsible for most cases of HAT [2]. In contrast to savannah tsetse (Morsitans species group) which respond strongly to odour cues, riverine flies characteristically respond weakly [3]. Effective odour cues for attracting riverine tsetse may yet be identified [4], but at present odourless, insecticide-treated cloth panels are advocated for the cost-effective control of these flies [2,5,6]. Understanding the visually-guided behaviours that draw tsetse to such baits can contribute to current efforts to optimise the cost and efficiency of control operations, and one factor that has received much attention is the role of colour [7,8,9,10].

Studies to understand tsetse attraction to baits have often employed grids of electrocuting wires which can enclose simple panels of coloured cloth bait material (e-cloths), or of fine net (e-nets). E-cloths sample tsetse that land on the cloth bait, whilst e-nets are difficult for tsetse to detect and sample those flies that accidentally collide with them [11,12,13]. This allows tsetse to be sampled not only when they contact a particular bait but also when circling nearby, allowing sophisticated investigation of their behaviour (e.g. [14]). As a result, it is recognised that tsetse are intercepted at baits as a function both of their initial attraction to approach the bait from a distance, and their propensity to land on the bait (or enter a trap) once close (e.g. [15]). A variety of interacting olfactory and visual cues can contribute to these behavioural processes (for reviews, [16,17]), but among them reflected light wavelength cues are both important, and relevant to the optimisation of the visual baits currently advocated for riverine tsetse control.

**Fig 1:**
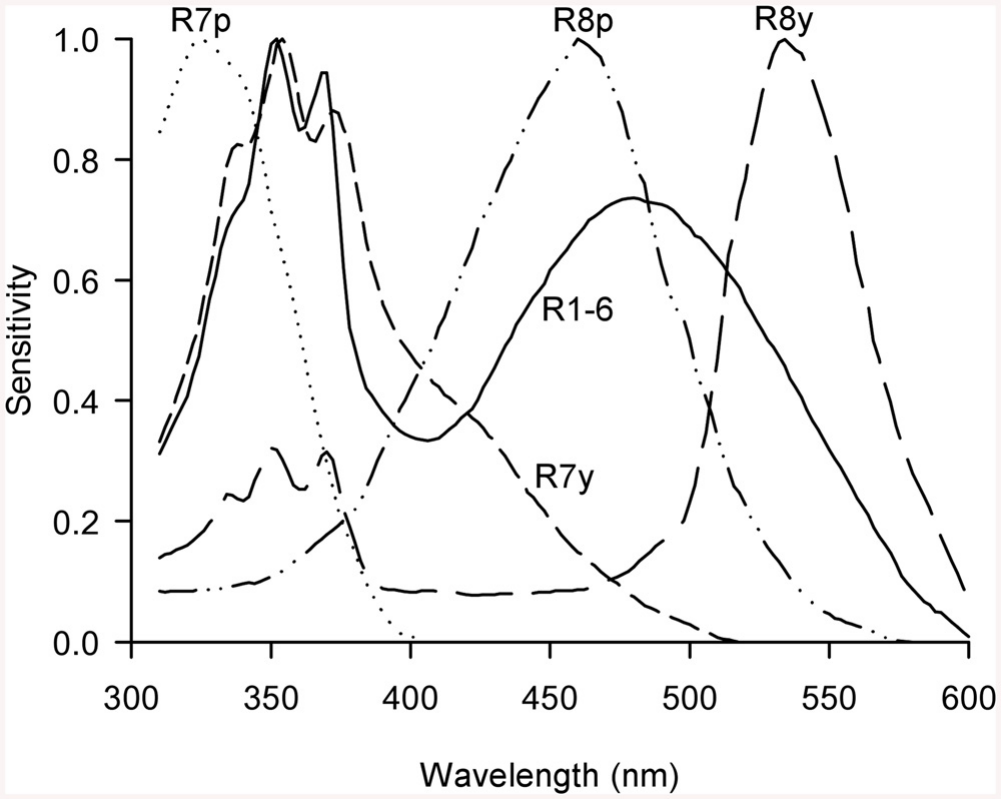
Fly photoreceptor sensitivity functions. Flies possess five classes of photoreceptor over the majority of the ommatidia in their compound eyes [18]. The typical spectral sensitivity functions of each photoreceptor class have been thoroughly characterised in *Musca* and *Calliphora*, and are shown here using data obtained from [18]. These functions were used by the author to calculate photoreceptor excitation by e-cloth reflectance spectra in a previous study [10]. This figure was produced by the author, and originally published in [10].

The role of colour cues in enticing tsetse to approach a stationary visual bait is relatively well understood, and the phthalogen blue dye for cotton fabrics produces a particularly attractive colour (e.g. [9]). Field studies monitoring combined tsetse catches at coloured e-cloths and flanking e-nets (sampling tsetse landing on the coloured cloth, and those circling it), have found positive contributions of blue wavelengths, and negative contributions of green/yellow/red and UV wavelengths, to the tsetse catch [8,9]. The same trends were also found in studies of tsetse catches in three-dimensional traps of various designs, although these catches would have resulted both from attraction into the vicinity of the traps, and trap entry responses [7,8]. The above insights were gained by direct analysis of visual bait reflectance spectra, but it is the responses of photoreceptors to these spectra that guide a fly’s behaviour. Across the majority of ommatidia in the fly compound eye, excluding the male fovea and the polarisation-sensitive dorsal marginal area, there are five classes of photoreceptor with varying spectral sensitivities (Fig 1) [18,19,20]. Recently, the datasets produced during the above tsetse field studies have been reanalysed using the calculated excitations of fly photoreceptors as predictors of attraction. The result of this reanalysis was that fly photoreceptors R7y (UV-blue) and R8p (blue) contribute positively, whilst R7p (shorter wavelength UV) and R8y (green) contribute negatively [10]. Perhaps because photoreceptors R7y and R8p provide somewhat redundant information, the attraction of tsetse to approach a visual bait, and the special attractiveness of phthalogen blue cotton, could be parsimoniously explained by a simple opponent mechanism involving the calculated excitations (*E*) of three of these photoreceptors as follows: *+E*_*R7y*_ *–E*_*R8y*_ *–E*_*R7p*_ [10].

**Fig 2:**
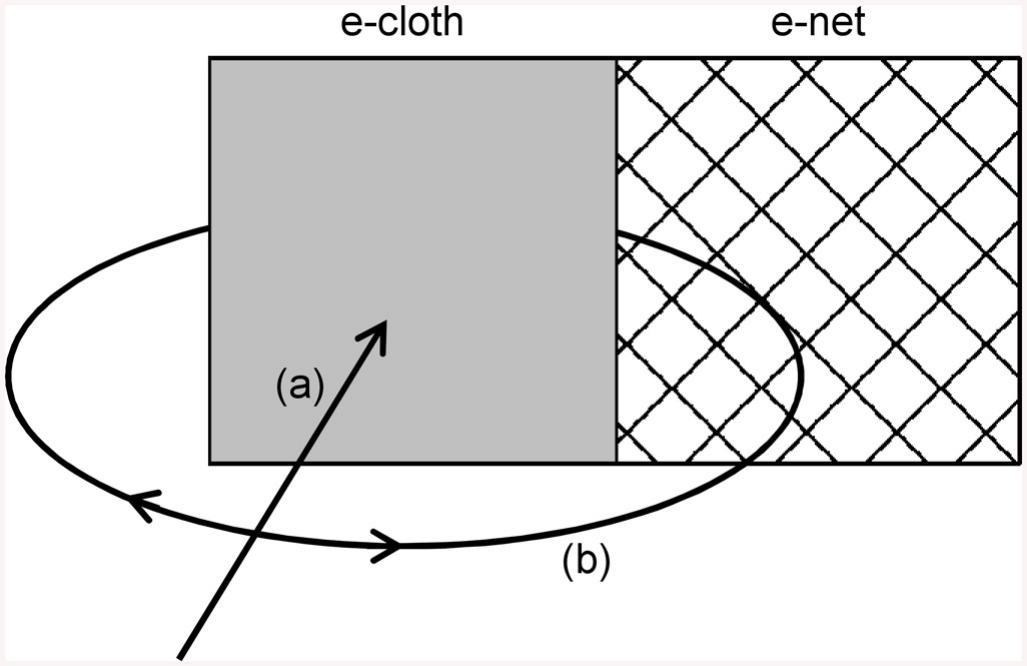
Measurement and interpretation of tsetse catch distribution between e-cloths and flanking e-nets. Arrows provide a schematic illustration of tsetse flight paths. (a) Tsetse attracted into the vicinity of an e-cloth may sometimes fly directly towards it and make contact. (b) More often, tsetse do not approach the e-cloth directly, but instead circle in its vicinity or alight nearby. Some of these flies make accidental contact with the e-net because it is difficult for them to detect. The tsetse catch of the e-cloth plus that of the e-net is known as the combined catch and is taken to indicate overall attraction to approach the visual bait. The proportion of the combined catch that was intercepted at the e-cloth is termed P_cloth_ in this study. This measurement indicates the prevalence of direct contact with the e-cloth over circling behaviour. For this reason, this measurement is often referred to as the ‘landing score’.

Videographic observations demonstrate that when tsetse alight on a black cloth target, they very rarely do so after having made a direct approach to it. Instead, the initial approach is followed by local circling or alighting on the ground before the fly eventually lands on the target [13]. This accords with data gained using combinations of e-cloth and flanking e-net, where the e-net sample of circling flies often exceeds the e-cloth sample of those that land directly [5,6,9]. Furthermore, intricate studies using e-nets reveal that only some of the flies attracted to a bait ultimately land at all, the others departing after having circled it [14,15]. Since the insecticide-treated cloth panels used for tsetse control can only be effective if tsetse make contact with them, insecticide-treated flanking nets are advocated to intercept and kill circling flies by inducing accidental collisions [5,6,21]. However, the cues that induce tsetse to alight remain an interesting and little understood area of investigation. Where field studies have employed combinations of e-cloth and flanking e-net, the catch of the e-cloth expressed as a proportion of the combined catch of the e-cloth and e-net (henceforth, P_cloth_) is used to provide a measurement of tsetse preference for direct landing over circling (see Fig 2). As such, this measurement is commonly referred to as the ‘landing score’. P_cloth_ is positively influenced by a bait’s reflectance of UV wavelengths, or low overall luminance, and the former observation has lead to the assertion that UV wavelengths are important cues for eliciting landing [8,15,22,23] (but see also [9]). Hence, a number of studies have investigated dual-colour baits, incorporating panels of colour that strongly stimulate tsetse to approach, and others that provide the putative landing cues (e.g. [22,24]).

The idea that landing responses are positively influenced by UV wavelengths appears to be at odds with the negative contribution of these wavelengths to the chromatic mechanism of attraction to the vicinity of the bait [7,8,9,10]. This would imply that a visual cue that is unattractive at long-range is attractive at close-range, and that entirely different behavioural mechanisms underlie visual attraction, broadly defined, at these different ranges. In this study I aim to shed light on this apparently paradoxical aspect of tsetse behaviour by providing a mechanistic explanation for P_cloth_ measurements based upon calculated excitation values for fly photoreceptors (c.f. [25,26,27,28]).

## Methods

### Catch distribution data

The distribution of tsetse catches between e-cloth and flanking e-net was analysed for two field datasets [8,9], out of the four recently analysed to determine a photoreceptor-based model of tsetse attraction [10]. These datasets were selected because they were obtained using simple, two-dimensional e-cloths of various colours, with adjacent two-dimensional e-nets, both oriented vertically (for a simplified schematic representation, see Fig 2).

Data for catches of *G*. *fuscipes fuscipes* at small e-cloths (0.25 m x 0.25 m) with equal-sized flanking e-nets were obtained from [9]. In total 37 cotton or polyester e-cloths of different colours were tested in 15 separate experiments. Each experiment investigated tsetse catches at five differently coloured e-cloths, one of which was always a phthalogen blue-dyed cotton standard. Phthalogen blue is often reported to be extremely attractive to tsetse, but the dye can only be applied to cotton fabrics [9]. The original study reported the proportion of the combined catch taken from the e-cloth (there termed the landing score), and absolute numbers of flies in the combined catch, for each e-cloth in each experiment. The number of landing flies was calculated from these data, rounding to the nearest whole number.

Data for catches of *G*. *palpalis palpalis* at large e-cloths (1.0 m x 1.0 m) with flanking e-nets (0.5 m x 1.0 m) were obtained from [8]. In total, 27 e-cloths of different colours were tested in 10 separate experiments. Where the type of fabric comprising the e-cloths was stated, it was reported to be cotton [8]. Each experiment investigated tsetse catches at four differently coloured e-cloths, one of which was always a phthalogen blue standard. The original study reported the percentage of the combined catch taken from the cloth panel (there termed the landing score) for each e-cloth in each experiment, although the absolute numbers of tsetse in the combined catch was stated only for the phthalogen blue standards.

### Calculated photoreceptor excitations

Fly photoreceptor excitation values elicited by each coloured e-cloth in the above tsetse field studies were calculated during a previous study [10]. That study made freely available in its supplementary materials the calculated excitation values and the materials required to calculate them, and completely described the calculation procedure (dx.doi.org/10.1371/journal.pntd.0003360) [10]. A brief recap of those methods is provided here for convenience.

Methods with which to calculate photoreceptor excitation from spectra of illumination, stimulus reflectance, background reflectance, and photoreceptor sensitivity are now well established and widely employed (e.g. [25,29]). For each fly photoreceptor type the effective quantum catch (P) of reflected light from a given e-cloth was calculated according to:

$$P=R\int_{310}^{600}I_{S}(\lambda )S(\lambda )D(\lambda )d\lambda$$

Where I_S_(λ) is the spectral reflectance function for the e-cloth; S(λ) is the spectral sensitivity function of the photoreceptor in question; and D(λ) is the illuminant function. R is the range sensitivity factor which adjusts photoreceptor sensitivity such that background stimulation would elicit a half maximal response in each receptor class, and was calculated by:

$$R=1/\int_{310}^{600}I_{B}(\lambda )S(\lambda )D(\lambda )d\lambda$$

Where I_B_(λ) is the spectral reflectance function of the assumed background.

Quantum catches were non-linearised to represent the transduction process in each photoreceptor, providing excitation (E) by:

E=P/(P+1)

Calculated photoreceptor excitations have values between 0.0 and 1.0, and through the above procedures the adapting background elicits a half-maximal response of 0.5 units in each photoreceptor [10,29].

The reflectance spectrum of a typical green leaf was used as the background reflectance spectrum, and the illuminant used was the D65 standard expressed as relative quanta (these are provided in S5 table). Both functions were obtained from [29], and were linearly interpolated to achieve 2 nm wavelength resolution. E-cloth reflectance spectra were obtained from the supplementary materials of [9], and linearly interpolated for 2 nm wavelength resolution, or extracted from figures in [8] using Datathief software [30] (the latter are provided in S5 table, whilst the former are freely available online at dx.doi.org/10.1371/journal.pntd.0001661). Photoreceptor sensitivity functions were those typical of *Musca* and *Calliphora* extracted from [18] using Datathief (see Fig 1). Although sensitivity functions have been recorded for *G*. *morsitans morsitans*, the flies used lacked carotenoid screening pigments due to dietary deficiency [19]. Carotenoid pigments were, however, extracted from the retinae of *G*. *p*. *palpalis* raised on a different diet [19]. The extent of visual screening in wild tsetse is thus unknown and would presumably vary with diet, but the underlying organisation of photoreceptors in tsetse aligns with that for *Musca* and *Calliphora* [18,19,31].

The approach taken in this study was to seek statistical explanations for landing scores based upon individual photoreceptor excitation values, and/or indices representing the combined responses of two or more photoreceptor types (c.f. [25,26,27,28]). One such combination was an opponent index representing the chromatic mechanism proposed to underlie attraction, calculated as follows: *+ E*_*R7y*_ *–E*_*R8y*_ *–E*_*R7p*_ [10]. This index was previously shown to predict combined e-cloth plus e-net catches in the *G*. *f*. *fuscipes* and *G*. *p*. *palpalis* datasets analysed here [10]. In order to aid in data interpretation, the opponent index was also calculated for leaves in the adapting background (+ 0.5–0.5–0.5 = -0.5).

### Statistical analyses

The two tsetse catch datasets each included a number of separate experiments in which sub-sets of e-cloths were compared, and these were often clustered around similar values of the opponent index. Therefore, I used Generalized Estimating Equations (GEEs) to try to model the clustering of data within experiments [32,33], without including ‘experiment’ as a factor in the analysis because this might have masked the overall relationship with opponent index or other predictors. The original experiments used latin squares designs to block out variation due to bait location and day, but the experiments themselves were separated in time. Thus, it was reasonable to expect that tsetse catches within each experiment would be related, but no particular structure was expected to the relatedness within experiment. As such, an exchangeable working correlation matrix was appropriate.

Because P_cloth_ is calculated from a known total number of flies in each combined catch, it is appropriate to analyse these measurements using a binary logistic model which correctly models the variance of such proportions [34]. This was possible for the *G*. *f*. *fuscipes* dataset where the total numbers of flies in each combined catch were directly reported. For these data a binomial distribution—logit link GEE model was employed. However, in the *G*. *p*. *palpalis* dataset absolute combined catches were reported only for the phthalogen blue cloth, with percentage catches for each of the other cloths within an experiment. The stated percentage catches were often not achievable by dividing any absolute catch integer value by that stated for the standard, presumably because the percentage catches were calculated from detransformed means as in other previous studies [9]. Hence, the numbers of flies in each combined catch could not be determined with certainty, and I decided instead to analyse P_cloth_ values directly, after logit transformation [34], using a normal distribution—identity link GEE model. Such approaches incorrectly assume equal variances across measured proportions, which can reduce their statistical power to detect differences [34]. Nevertheless, the distribution of the residuals from the normal—identity GEE analyses reported in the main text did not differ markedly from a normal distribution (as determined by Kolmogorov-Smirnov tests and visualisation of Q-Q plots), or demonstrate a strongly marked pattern when plotted against values for the linear predictor.

The goodness of fit of GEE models was assessed using the quasi-likelihood under independence model criterion (QIC), and a version of this statistic that corrects for model complexity and small sample size (QICC) [35,36]. QIC is a modification to Akaike’s information criterion (AIC) for use with GEE models [35], and lower values for such criteria indicate improved fit to the data. With respect to AIC, models within 2 units of the best model are sometimes considered to be competitive [37]. All analyses were conducted using SPSS version 22.0 (IBM Corp., Armonk NY, USA).

## Results

### Can catch distribution be explained by the mechanism proposed to underlie attraction?

**Fig 3:**
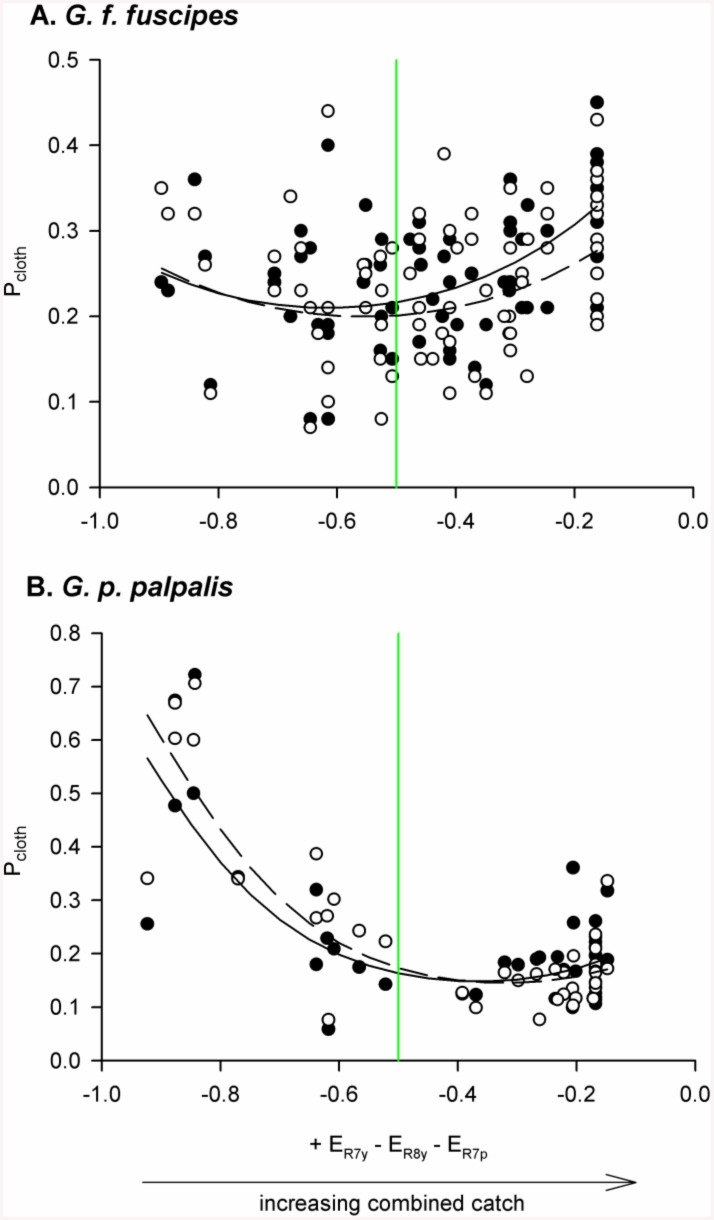
P_cloth_ was non-linearly related to an opponent index describing the mechanism of visual attraction. P_cloth_ values are plotted for male (filled circles) and female (open circles) *G*. *f*. *fuscipes* (A) and *G*. *p*. *palpalis* (B). Data for *G*. *f*. *fuscipes* come from a field study in which e-cloths and e-nets were both 0.25 m x 0.25 m [9], and those for *G*. *p*. *palpalis* from a field study in which e-cloths were 1.0 m x 1.0 m, and flanking e-nets 0.5 m x 1.0 m [8]. The x-axes of both plots display a calculated photoreceptor opponent index that approximates the previously reported mechanism of tsetse attraction to approach a visual bait, and with which combined catches of e-cloth and e-net were positively related in both field studies (this trend is illustrated by the horizontal arrow below panel B) [10]. The plotted relationships are detransformed logits obtained from the statistical analyses in Table 1. Vertical green lines indicate an opponent index calculated for the assumed background of green leaves.

The chromatic mechanism proposed to underlie tsetse attraction can be approximated by a simple opponent index, and the combined catch of an e-cloth and flanking e-net was previously shown to have a positive relationship with this index (see Fig 7 of [10]). Fig 3 shows the relationship between this same opponent index and P_cloth_ (the proportion of the combined catch that was caught on the e-cloth), which represents the propensity of tsetse to directly contact the cloth panel in preference to first, or only, circling around it (see Fig 2). In contrast to combined catches, P_cloth_ did not have a simple, positive relationship with opponent index. GEE models containing a quadratic term had lower QIC and QICC versus simpler linear models for all datasets (Table 1; Fig 3). However, whilst the fit of the quadratic model was substantially better than that of the linear model for *G*. *f*. *fuscipes*, the two models were competitive for *G*. *p*. *palpalis* (for which the linear model described a negative relationship between P_cloth_ and opponent index).

**Table 1:** GEE models evaluating the relationship between opponent index and P_cloth_.

Dataset Model		*G*. *f*. *fuscipes*		*G*. *p*. *palpalis*	
		Linear	Quadratic	Linear	Quadratic
**Males**					
Intercept	B	-0.576	-0.201	-1.885	-0.873
	Wald X^2^_1_	42.758	2.309	105.699	11.206
	(p)	**(<0.001)**	**(0.129)**	**(<0.001)**	**(0.001)**
Opp. Index	B	1.216	3.652	-1.470	4.775
	Wald X^2^_1_	23.955	28.666	5.080	8.436
	(p)	**(<0.001)**	**(<0.001)**	**(0.024)**	**(0.004)**
Opp. index^2^	B		2.965		6.507
	Wald X^2^_1_		20.993		9.875
	(p)		**(<0.001)**		**(0.002)**
QIC		32066.345	**28572.492**	24.512	**24.131**
QICC		32042.821	**28554.126**	18.947	**16.845**
**Females**					
Intercept	B	-0.875	-0.533	-2.091	-1.100
	Wald X^2^_1_	57.165	11.556	218.304	19.063
	(p)	**(<0.001)**	**(0.001)**	**(<0.001)**	**(<0.001)**
Opp. Index	B	0.818	3.085	-2.009	4.203
	Wald X^2^_1_	9.731	13.494	14.097	5.300
	(p)	**(0.002)**	**(<0.001)**	**(<0.001)**	**(0.021)**
Opp. index^2^	B		2.780		6.550
	Wald X^2^_1_		9.139		9.996
	(p)		**(0.003)**		**(0.002)**
QIC		53417.982	**49664.141**	20.424	**19.843**
QICC		53382.620	**49629.109**	16.066	**14.128**

Significant effects (p<0.05), and the lowest (best fitting) QIC and QICC values, are highlighted using bold font.

The green vertical line in each panel of Fig 3 shows the opponent index value calculated for leaves in the adapting background, to which each photoreceptor responds with a half-maximal response of 0.5 units of excitation [10,29]. The fitted quadratic relationships suggest that P_cloth_ tended to increase with opponent index for visual baits that were more attractive than their background, although this trend was much more marked for *G*.*f*. *fuscipes* than for *G*. *p*. *palpalis*. Such a trend might be expected if the mechanism implicated in initial attraction also underlay landing responses (Fig 3, to the right of the green lines). However, inconsistent with this explanation, the fitted quadratic relationships also tended to increase as visual baits became increasingly less attractive than their background, although in this respect the trend was more marked for *G*.*p*. *palpalis* than *G*. *f*. *fuscipes* (Fig 3, to the left of the green lines). These quadratic relationships between P_cloth_ and opponent index were not considered to be biologically meaningful in themselves, but were hypothesised to be evidence that a second behavioural mechanism interacts with opponent index in determining tsetse catch distribution.

### The interaction of two behavioural mechanisms explains catch distribution

**Fig 4:**
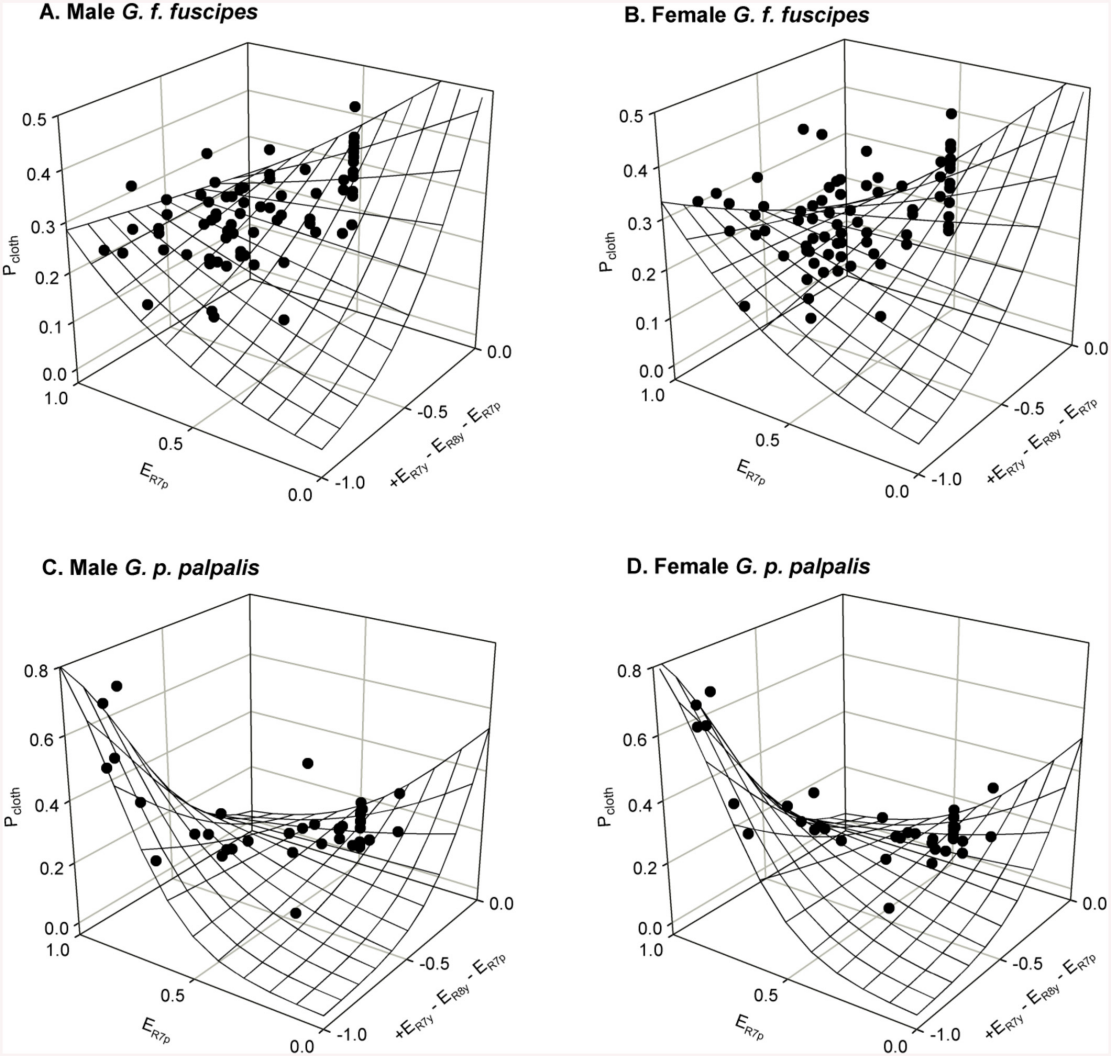
P_cloth_ plotted as a function of opponent index describing attraction, and an interacting photoreceptor R7p-driven mechanism. P_cloth_ values are those from Fig 3, plotted separately for male and female *G*. *f*. *fuscipes* (A and B), and for male and female *G*. *p*. *palpalis* (C and D). The overall trend across datasets was for an increase in P_cloth_ with increases in the opponent index that explains overall attraction; and an increase in P_cloth_ with increases in the strength of the photoreceptor R7p response. Plotted grids represent the regression planes statistically tested in Tables 2 and 3, and are the detransformed logits obtained from those linear relationships.

I next conducted GEE analyses to model P_cloth_ based upon the opponent index describing visual attraction, excitation values of photoreceptors that may drive a second behavioural mechanism, and the interaction between these two mechanisms (Tables 2 and 3). With the exception of the model containing photoreceptor R8p excitation for the female *G*. *f*. *fuscipes* dataset, all of these models resulted in reductions in QIC and QICC over the linear relationships with opponent index alone presented in table 1. Of these models, that which used the shorter wavelength UV photoreceptor R7p’s response consistently fitted each dataset better than models using excitation values for any other photoreceptor type, and in the R7p models the effects of all predictors were significant (Tables 2 and 3; Fig 4). Judged by differences in QIC >2, no other model was deemed competitive with the R7p model, although for the *G*. *p*. *palpalis* dataset QICC differences <2 provided some support for the alternative models other than that using R8p excitation. Removing the interaction term from any R7p model reduced its fit to the data. Elaborating any R7p model with an additional photoreceptor excitation value and its interaction term also reduced its fit to the data (S1 and S2 tables).

**Table 2:** GEE models explaining *G*. *f*. *fuscipes* catch distribution based upon attraction opponent index, and an interacting mechanism driven by one of the five photoreceptor types individually.

R[#]		R7p	R7y	R1-6	R8p	R8y
**Males**						
Intercept	B	0.306	0.314	-0.068	-0.305	0.011
	Wald X^2^_1_	1.320	0.416	0.041	1.124	0.002
	(p)	(0.251)	(0.519)	(0.839)	(0.289)	(0.967)
Opp. Index	B	3.927	3.957	2.953	2.212	3.052
	Wald X^2^_1_	10.458	6.291	7.341	5.487	16.524
	(p)	**(0.001)**	**(0.012)**	**(0.007)**	**(0.019)**	**(<0.001)**
R[#]	B	-1.582	-1.248	-0.746	-0.367	-1.190
	Wald X^2^_1_	9.042	3.667	2.817	1.120	6.000
	(p)	**(0.003)**	(0.056)	(0.093)	(0.290)	**(0.014)**
Interaction	B	-4.284	-3.677	-2.459	-1.338	-3.279
	Wald X^2^_1_	10.975	3.790	3.583	1.634	9.228
	(p)	**(0.001)**	(0.052)	(0.058)	(0.201)	**(0.002)**
QIC		**27724.357**	28887.066	29664.800	30664.424	28838.041
QICC		**27700.435**	28863.876	29644.809	30643.027	28820.388
**Females**						
Intercept	B	0.175	0.401	-0.307	-0.552	-0.175
	Wald X^2^_1_	0.284	0.445	0.584	2.135	0.353
	(p)	(0.594)	(0.505)	(0.445)	(0.144)	(0.552)
Opp. Index	B	3.641	3.440	2.017	1.319	2.242
	Wald X^2^_1_	8.463	3.220	2.537	1.340	7.233
	(p)	**(0.004)**	(0.073)	(0.111)	(0.247)	**(0.007)**
R[#]	B	-1.996	-1.886	-0.903	-0.470	-1.681
	Wald X^2^_1_	7.954	4.360	1.978	0.702	5.962
	(p)	**(0.005)**	**(0.037)**	(0.160)	(0.402)	**(0.015)**
Interaction	B	-4.770	-3.764	-1.819	-0.710	-3.092
	Wald X^2^_1_	9.095	2.169	1.067	0.219	4.920
	(p)	**(0.003)**	(0.141)	(0.302)	(0.640)	**(0.027)**
QIC		**47265.969**	50577.830	52790.299	53833.444	51761.238
QICC		**47225.233**	50538.370	52751.289	53793.631	51722.320

Significant effects (p<0.05), and the lowest (best fitting) QIC and QICC values, are highlighted using bold font. The model containing R7p excitation appeared to provide the best fit to the data.

**Table 3:** GEE models explaining *G*. *p*. *palpalis* catch distribution based upon attraction opponent index, and an interacting mechanism driven by one of the five photoreceptor types individually.

R[#]		R7p	R7y	R1-6	R8p	R8y
**Males**						
Intercept	B	0.148	-0.282	0.105	-0.435	-0.022
	Wald X^2^_1_	0.201	1.161	0.067	1.672	0.002
	(p)	(0.654)	(0.281)	(0.795)	(0.196)	(0.961)
Opp. Index	B	5.951	4.636	6.714	4.297	5.852
	Wald X^2^_1_	104.809	10.570	13.642	5.631	13.657
	(p)	**(<0.001)**	**(0.001)**	**(<0.001)**	**(0.018)**	**(<0.001)**
R[#]	B	-3.465	-2.276	-2.783	-2.041	-3.092
	Wald X^2^_1_	15.432	22.605	19.147	16.283	13.270
	(p)	**(<0.001)**	**(<0.001)**	**(<0.001)**	**(<0.001)**	**(<0.001)**
Interaction	B	-10.676	-8.089	-10.753	-7.652	-10.495
	Wald X^2^_1_	89.160	18.744	20.090	10.619	20.276
	(p)	**(<0.001)**	**(<0.001)**	**(<0.001)**	**(0.001)**	**(<0.001)**
QIC		**15.525**	19.896	18.417	21.162	19.991
QICC		**14.688**	16.370	16.579	17.670	17.041
**Females**						
Intercept	B	0.021	-0.890	-0.363	-0.947	-0.551
	Wald X^2^_1_	0.006	12.913	0.753	8.998	1.148
	(p)	(0.936)	**(<0.001)**	(0.386)	**(0.003)**	(0.284)
Opp. Index	B	5.174	3.796	6.468	3.784	5.529
	Wald X^2^_1_	50.618	10.873	13.017	5.061	8.039
	(p)	**(<0.001)**	**(0.001)**	**(<0.001)**	**(0.024)**	**(0.005)**
R[#]	B	-3.664	-1.588	-2.169	-1.485	-1.987
	Wald X^2^_1_	42.200	16.531	13.101	12.352	5.234
	(p)	**(<0.001)**	**(<0.001)**	**(<0.001)**	**(<0.001)**	**(0.022)**
Interaction	B	-10.429	-7.540	-10.833	-7.566	-10.000
	Wald X^2^_1_	107.792	31.449	24.757	14.454	15.200
	(p)	**(<0.001)**	**(<0.001)**	**(<0.001)**	**(<0.001)**	**(<0.001)**
QIC		**11.465**	15.835	14.359	16.812	16.654
QICC		**12.279**	13.730	13.395	14.596	14.255

Significant effects (p<0.05), and the lowest (best fitting) QIC and QICC values, are highlighted using bold font. The model containing R7p excitation appeared to provide the best fit to the data.

To further support the adequacy of the opponent index/R7p model, I also computed sums of, and differences between, the excitation values of every possible combination of photoreceptor pairs and used these in GEE models that also contained opponent index and an interaction term (S3 table). Alongside opponent index, summed excitation values of photoreceptor pairs (representing an additional achromatic mechanism) generally fitted the data better than computed differences between the excitation values of photoreceptor pairs (representing an additional chromatic mechanism). In the *G*. *f*. *fuscipes* datasets, the model including summed R7p and R7y excitations alongside opponent index was the only one for which there was a substantial improvement in QIC or QICC over the opponent index/R7p model, but this was evident only for males and not for females (S3 table). In the *G*. *p*. *palpalis* datasets, many summed photoreceptor models provided largely equivalent QIC or QICC values (i.e. within 2 units) to the opponent index/R7p model, but among these reductions were only evident in QICC, and only for models in which R7p excitation was part of the photoreceptor sum (S3 table).

In order to rule out the potentially simpler possibility that P_cloth_ might result entirely from a single achromatic or chromatic mechanism, I examined GEE models containing every possible combination of between one and five photoreceptor types to predict P_cloth_ (S4 table). These models fitted the data substantially less well than the above models with two interacting mechanisms, indicating that they did not provide a better explanation for tsetse behaviour. Thus, overall, these analyses support the assertion that P_cloth_ can be predicted by the colour opponent model that was proposed to underlie initial attraction, and an additional, interacting achromatic mechanism reliant on excitation from photoreceptor R7p (Fig 4). However, the additional contribution of other photoreceptors to that achromatic mechanism should not be ruled out.

## Discussion

In this study I reanalysed tsetse catch distribution across coloured e-cloths and flanking e-nets based upon a chromatic mechanism recently proposed to explain tsetse attraction to approach visual baits. I found that P_cloth_ increased as cloth panels became more attractive by an index describing this mechanism, as expected if the same chromatic mechanism of attraction underlay both the approach to a bait, and subsequent landing upon it. However, I also found that P_cloth_ increased as excitation of the UV-sensitive photoreceptor R7p increased, indicating that tsetse are also driven to contact cloth panels as a result of a separate but interacting achromatic mechanism.

It seems intuitive that tsetse should more readily alight upon cloth panels that are more attractive by the chromatic mechanism implicated in their initial attraction to approach them. However, the involvement of a second, achromatic mechanism in causing tsetse to directly contact such cloth panels is less easy to explain. Flies are well-known to display an innate attraction to UV light and in *Drosophila* the R7 photoreceptors are important in driving this response [38,39]. This behaviour is often called the ‘open space response’, and is presumed to guide flies towards areas of open sky. This is because the sky is strongly radiant in UV wavelengths, whilst many features of the terrestrial environment are characterised by strong UV absorption (e.g. see [40]). Earlier tsetse work has already suggested that UV wavelengths may functionally represent skylight, causing highly UV-reflective cloth panels to elicit high P_cloth_ values not by eliciting landing responses, but as a result of accidental collisions by tsetse attempting to disperse [15,23]. The R7p-driven achromatic mechanism suggested by my analysis appears well aligned with these explanations, which would suggest that tsetse catch distributions are affected by two distinct behavioural motivations. In further support of this idea, *G*. *tachinoides* caught on e-cloths tended to have lower fat content than those caught on flanking e-nets, which was interpreted as an indication that the relatively more starved flies were more prone to land directly in preference to circling, due to their requirement to be less discriminating in host seeking [24]. In the same study, female flies caught over the UV-reflective white portion of a half-blue, half-white e-cloth had higher fat content than those caught over the blue portion, and their fat content was equivalent to that of flies caught at flanking e-nets of other target designs in the same experiment [24]. This trend was, however, not evident for males. Nevertheless, since highly UV-reflective baits are unattractive to host-seeking tsetse [7,8,9,10], the fact that better-nourished and potentially more discriminating female flies tended to make contact with them [24], would be consistent with the explanation that these flies were attempting to disperse rather than land on a perceived host. However, detailed observations of tsetse behaviour prior to interception on UV- and non-UV-reflective cloth panels, as have been made of tsetse behaviour prior to alighting on black panels [13], will be required to directly test this hypothesis and provide persuasive evidence for the above explanation.

A UV effect on P_cloth_ was not evident in the authors’ original analysis of the *G*. *f*. *fuscipes* dataset [9], and in this reanalysis the analogous R7p effect was notably weaker than that seen for *G*. *p*. *palpalis*. A plausible explanation for this difference between datasets is the different size of the e-cloths in the two studies: those in the *G*. *f*. *fuscipes* study were 1/16^th^ the size of those in the *G*. *p*. *palpalis* study. Alighting responses of savannah tsetse increase with the size of blue or black targets [41,42], whilst the alighting responses of riverine species are relatively little affected by changes in the size of such a target [5]. A potential explanation for this is the effect of habitat geometry on tsetse movement and expression of host-seeking behaviour [43]. However, if some of the tsetse intercepted by UV-reflecting baits are in fact attempting to orient towards perceived open spaces rather than alighting on perceived hosts, it is plausible that the larger area of those open spaces enhanced this separate behavioural response, resulting in the difference between the datasets. However, a number of other explanatory factors cannot be ruled out. The UV effect was clearly evident in a study of the riverine tsetse *G*. *p*. *palpalis* [8], but only for a sub-set of UV-reflective baits which also allowed some light to pass through them in a study of the savannah tsetse *G*. *pallidipes* [23]. It is certainly possible that species differences in behaviour explain such discrepancies, but it must also be borne in mind that the highly UV-reflective baits that elicit high P_cloth_ values also tend to attract the lowest combined catches, resulting in greater error around P_cloth_ measurements for such baits. This factor has special relevance to the current analysis, since the binomial—logit GEE model applied to *G*. *f*. *fuscipes* data correctly modelled the variance of P_cloth_ measurements, whilst this was not true of the normal—linear GEE model that was applied to logit-transformed *G*. *p*. *palpalis* P_cloth_ values for reasons of data availability. For this reason, some caution should be exercised in evaluating the trends for *G*. *p*. *palpalis*, although trends in that dataset were strongly evident, and substituting binary logistic models for linear ones might be expected to reduce statistical power [34].

An additional factor that might cause variation in the UV effect on P_cloth_ is the specific positioning of a visual bait. This might lead to variability in the effect of colour cues on attraction and landing as a result of variation in the background they are viewed against, or their spectrum of illumination. In possible support of this general notion, a study of *G*. *tachinoides* in Cote d’Ivoire found significant differences in attraction to blue, violet, red, and black e-cloths between replicates conducted in gallery forest, and those conducted on more open riverbank habitat [24]. Furthermore, P_cloth_ was significantly higher for males in the riverbank replicate. However, whilst this supports the general notion that bait positioning may be an important factor affecting visual cues and behavioural responses to them, the same study provided no evidence that such factors might affect the UV effect on P_cloth_ specifically. This was because there were no apparent differences between replicates of an experiment incorporating high- and low-UV reflectance white baits in the same two habitats, and the UV effect on P_cloth_ was only evident for females in the combined data from both replicates [24]. An additional way in which bait positioning may affect P_cloth_ is via active avoidance of e-nets, which has been shown to be greater in shade than full sun [13]. Active avoidance of the e-net would cause an increase in P_cloth_, as a result of a reduction in combined catch. Finally, other cues which were not quantified in the original field studies may also influence landing responses. For example, polarotaxis has been implicated in attraction and landing of tabanid flies on potential hosts and artificial baits [44,45,46], but the visual baits analysed in this study were not quantified with respect to reflected polarised light.

Alongside the R7p-driven achromatic mechanism, this study also provides evidence that the chromatic mechanism guiding tsetse attraction towards a visual bait might also encourage them to land upon it. This suggests that the same mechanism underlies attraction at both long- and close-range. By comparison with findings for plant-seeking insects (e.g. [28]) it was argued that blue-green (R7y-R8y) opponency provides a means to distinguish vegetation from other objects, such as potential vertebrate hosts [10], which aligns with previous explanations for the blue preference of tsetse [16]. The additional, negative input of photoreceptor R7p improved the fit to the data, and was thus implicated in the opponent mechanism underlying attraction [10]. Given the above interpretation of the functional role of R7p and UV wavelengths, this input may function to distinguish patches of open sky from vegetation and potential hosts. However, in light of the analyses presented in this study, it might be debated whether R7p’s effect on attraction comes about as a result of its input to the proposed chromatic mechanism, or solely via the interaction of the achromatic mechanism suggested here.

Low luminance black fabrics are also well known to elicit strong tsetse landing responses (e.g. [15,42]). Such fabrics are characterised by low reflectance at all wavelengths, including the UV, so these landing responses cannot be explained by the R7p-driven achromatic mechanism suggested here. The opponent index used to describe attraction in this analysis simply subtracts the excitation of photoreceptors R7p and R8y from that of R7y, and as a result the value is negative for all stimuli in this analysis with those closest to zero the most attractive. Because black fabrics have uniformly low luminance, they elicit low excitation values in all photoreceptors, and as a result of that also have opponent indices that are relatively close to zero and, therefore, are predicted to be attractive [10]. With the important caveat that neural computations in a fly’s brain will differ to a greater or lesser extent from their simplified representation here, the ability of black fabrics to elicit tsetse landing responses is compatible with the scheme described in this analysis. However, other explanations must not be ruled out, such as a separate role for low luminance in attraction, or the involvement of polarotaxis for which dark surfaces are particularly effective in providing polarised light cues [20,46]. Studies of a range of *Glossina* species have reported decreased catches using blue/black combination e-cloths, when the cloth panels inside the electrocuting grids were covered by an adhesive sheet that absorbed UV wavelengths [47,48,49]. This resulted from decreased tsetse catch over the black portion of the cloth panel only. In these studies the UV reflectance of the black cloth was low, meaning that this result is unlikely to be explained by an effect of the UV manipulation on the R7p mechanism described in the current analysis. Since the adhesive film absorbed wavelengths below 400 nm [49], it would have affected not only the repellent R7p response (shorter wavelength UV), but also the attractive R7y response (UV-blue), and may thus have had complex effects on the mechanism of attraction. It is also possible that the adhesive sheet affected other visual cues, such as the polarisation of reflected light [44,45,46].

Intercepting circling tsetse has great potential to augment catches since the majority of the tsetse attracted into the vicinity of a bait circle around it rather than landing [6,9,14,15]. This has motivated the use of insecticide-treated flanking nets to intercept circling flies, and these are important additions to the small cloth panels currently advocated for riverine tsetse control, where their small size and the use of modern netting materials make them robust [5,6]. By contrast, larger visual baits are employed for savannah tsetse, and large flanking nets to accompany these have sometimes been suggested to be damage prone [5,6]. However, although flanking net damage did reduce a bait’s efficacy in field trials, replacement rates were higher for net than cloth portions but low in both cases (0.2 versus 0.1% monthly replacement rate, respectively) [50]. Furthermore, savannah tsetse landing responses increase with bait size [41,42,51], such that large cloths can function just as efficiently as cloth and flanking net combinations of the same size [42]. Therefore, although some riverine tsetse may mistake highly UV-reflective cloths for patches of open sky, even if this finding were transferable to savannah tsetse it is unlikely to mean that UV-reflective cloths can provide a useful substitute for the flanking net. Nevertheless, the suggestion that UV-reflecting cloths likely catch tsetse attempting to disperse rather than host-seek does have implications for visual bait optimisation. Short wavelength excitation of photoreceptor R7p was previously shown to contribute negatively to the chromatic mechanism of attraction [10], and in the current analysis strong excitation of R7p was implicated as interacting with that mechanism. As such, the attractiveness of visual baits is likely best enhanced by reducing UV reflectance. The currently preferred phthalogen blue dye has these properties, but can only be applied to cotton material (e.g. [9]). Modern polyester fabrics offer a number of advantages in terms of cost and robustness, but the blues currently produced for tsetse control have broader reflectance peaks than phthalogen blue that extend into the UV (e.g. see reflectance spectra for blues 7 and 8 in [9]). Curtailing reflectance at low wavelengths and enhancing it in the attractive region using fluorescent dyes, as has been suggested previously [8], may be the key to optimising these fabrics. In addition, the use of stand-alone insecticide-treated, UV-reflective cloth panels without flanking nets might potentially provide a useful complement to the standard baits, if they do indeed attract a different sub-set of the tsetse population.

## Supporting Information

S1 Table GEE models explaining P_cloth_ for *G*. *f*. *fuscipes* based upon attraction opponent index, R7p photoreceptor excitation, and the excitation of an additional photoreceptor type. Table shows the Wald X^2^ statistic for each predictor, with its p value in brackets, and QIC and QICC values for each model. No additional photoreceptor type resulted in an improvement in fit to the data over the opponent index and R7p model from Table 2 and Fig 4, as judged by QIC and QICC.
(DOCX)

S2 Table GEE models explaining P_cloth_ for *G*. *p*. *palpalis* based upon attraction opponent index, R7p photoreceptor excitation, and the excitation of an additional photoreceptor type. Table shows the Wald X^2^ statistic for each predictor, with its p value in brackets, and QIC and QICC values for each model. No additional photoreceptor type resulted in an improvement in fit to the data over the opponent index and R7p model from Table 3 and Fig 4, as judged by QIC and QICC.
(DOCX)

S3 Table GEE models explaining P_cloth_ based upon attraction opponent index, and the sum or difference in excitation values of every possible photoreceptor pair. Some of the sums involving photoreceptor R7p provided an improvement in QIC and/or QICC over the model shown in Tables 2 and 3 and Fig 4 (green cells), but these trends were inconsistent across datasets.
(XLSX)

S4 Table GEE models explaining P_cloth_ based upon every possible combination of one to five photoreceptor excitations. None of these models resulted in an improvement in QIC or QICC over the model illustrated in Tables 2 and 3 and Fig 4. This indicates that any single mechanism underlying landing scores did not describe the data better than the two interacting mechanisms described in text.
(XLSX)

S5 Table Collated dataset. (XLSX)
