# Supplementary material for: A Receptor-Based Explanation for Tsetse Fly Catch Distribution between Coloured Cloth Panels and Flanking Nets
Source: PLoS Negl Trop Dis. 2015 Oct 16;9(10):e0004121. doi: 10.1371/journal.pntd.0004121 (PMC4608566; doi:10.1371/journal.pntd.0004121)
Supplement: S2 Table — Table shows the Wald X2 statistic for each predictor, with its p value in brackets, and QIC and QICC values for each model. No additional photoreceptor type resulted in an improvement in fit to the data over the opponent index and R7p model from Table 3 and Fig 4, as judged by QIC and QICC. (DOCX) [file pntd.0004121.s002.docx]

**S2 table: GEE models explaining P_cloth_ for *G. p. palpalis* based upon attraction opponent index, R7p photoreceptor excitation, and the excitation of an additional photoreceptor type.**

| **R[#]** | **R7y** | **R1-6** | **R8p** | **R8y** |
| --- | --- | --- | --- | --- |
| **Males** |  |  |  |  |
| Intercept | 1.075 (0.300) | 1.521 (0.217) | 1.197 (0.274) | 1.017 (0.313) |
| Opp. Index | 129.820 **(<0.001)** | 62.127 **(<0.001)** | 112.619 **(<0.001)** | 34.712 **(<0.001)** |
| R7p | 8.949 **(0.003)** | 5.960 **(0.015)** | 9.629 **(0.002)** | 3.178 (0.075) |
| R7p*Opp. Index | 46.774 **(<0.001)** | 29.770 **(<0.001)** | 50.067 **(<0.001)** | 17.916 **(<0.001)** |
| R[#] | 3.462 (0.063) | 3.520 (0.061) | 4.091 **(0.043)** | 1.943 (0.163) |
| R[#]*Opp. Index | 4.147 **(0.042)** | 3.507 (0.061) | 4.281 **(0.039)** | 1.561 (0.211) |
| QIC | 17.943 | 18.303 | 17.894 | 19.418 |
| QICC | 18.248 | 18.124 | 18.201 | 18.084 |
| **Females** |  |  |  |  |
| Intercept | 0.170 (0.680) | 0.216 (0.642) | 0.165 (0.685) | 0.002 (0.963) |
| Opp. Index | 73.661 **(<0.001)** | 58.776 **(<0.001)** | 70.939 **(<0.001)** | 35.894 **(<0.001)** |
| R7p | 29.367 **(<0.001)** | 24.839 **(<0.001)** | 28.503 **(<0.001)** | 16.068 **(<0.001)** |
| R7p*Opp. Index | 34.122 **(<0.001)** | 36.571 **(<0.001)** | 45.724 **(<0.001)** | 32.480 **(<0.001)** |
| R[#] | 0.678 (0.410) | 1.750 (0.186) | 1.352 (0.245) | 0.045 (0.832) |
| R[#]*Opp. Index | 11.841 **(0.001)** | 7.675 **(0.006)** | 10.688 **(0.001)** | 1.441 (0.230) |
| QIC | 15.738 | 15.843 | 15.637 | 16.569 |
| QICC | 15.500 | 15.537 | 15.655 | 15.656 |

Wald Χ^2^ is stated for each predictor in the model, with its associated significance level in brackets.
